# Stress load of Chinese nurses in Fangcang Shelter Hospitals during the COVID-19 pandemic: A latent profile analysis

**DOI:** 10.3389/fpubh.2022.1048358

**Published:** 2023-01-10

**Authors:** Liyan Gu, Jian Chang, Ji Wang, Ping Feng, Hao Xu

**Affiliations:** ^1^Department of Neurology, 905th Hospital of the PLA Navy, Shanghai, China; ^2^Nursing Department, Shanghai General Hospital, School of Nursing, Shanghai Jiao Tong University, Shanghai, China; ^3^Nursing Department, 905th Hospital of the PLA Navy, Shanghai, China; ^4^Nursing Department, Changhai Hospital, Naval Medical University, Shanghai, China; ^5^Department of Infectious Diseases, Changhai Hospital, Naval Medical University, Shanghai, China

**Keywords:** stress load, Fangcang Shelter Hospital, latent profile analysis, COVID-19, work-related stressors

## Abstract

The Omicron wave of the COVID-19 pandemic significantly affected Shanghai, China, from March to June 2022. Numbers of Fangcang Shelter Hospitals (FSHs) were conversed from stadiums and exhibition centers to tackle the pandemic. This study aimed to identify the stress load profiles of nurses working in FSHs and explore the characteristics and factors influencing stress load profiles. Totally, 609 out of 700 FSH nurses (with an effective response rate of 87%) participated in an online survey investigating their socio-demographic information, work-related stressors, and stress load. Results of the latent profile analysis identified four classes of stress load, which were labeled as the low (Class 1), mild (Class 2), moderate (Class 3), and high (Class 4) stress load class. Maternity status and self-perceived health condition were significantly different between the four stress load classes by comparisons using the Chi-square test and the Kruskal–Wallis test. The contributors to the stress load profiles were determined by the multinomial logistic regression analysis, including age, education, maternity status, self-perceived health condition, working time in FSHs, and the four dimensions of work-related stressors. Participants who were less healthy (OR = 0.045, 95% CI:0.012,0.171), worked longer time in FSHs (OR = 40.483, 95% CI: 12.103,135.410), faced with more workload (OR = 3.664, 95% CI: 1.047,12.815), and worse working environment (OR = 12.274, 95% CI: 3.029,49.729) were more likely to be classified to the high stress load class. The task arrangement and working environment for FSH nurses should be optimized, and psychological training should be conducted routinely.

## 1. Introduction

The COVID-19 pandemic was classified by the World Health Organization as an international event of concern, with rapid transmission, widespread infection, and difficulty in prevention and control. The Omicron wave of the pandemic has been observed worldwide, with the highest number of confirmed cases exceeding 580,000 per day (1, 2). The pandemic posed a massive threat to the physical and mental health of the public (3). In the face of the outbreak, China classified the novel coronavirus pneumonia (NCP) as a class B infectious disease and implemented prevention and control measures toward NCP with the standard of class A infectious diseases. In contrast with other nations, the Chinese government is strongly dedicated to the “dynamic zero” approach (4).

At the peak of the pandemic in Shanghai, there were more than 20,000 new cases per day during the Omicron wave from Mach to June 2022 (5). Within a short time, the capacity of medical services was stunned. According to the target requirements of “timely detection, rapid disposal, precise control, and effective treatment” and the strict implementation of the “early detection, early reporting, early isolation, early treatment” principle (6), Shanghai started to build two levels (municipal-district) of Fangcang Shelter Hospitals (FSHs) of different sizes, the total number of which had reached more than 110, with more than 250,000 running beds. FSHs, often called cabin hospitals, are frequently employed in large-scale disasters due to their speedy construction, enormous scale, and low cost to accommodate emergency medical rescue missions (7). To combat the COVID-19 pandemic, they have lately been widely adopted in China by transforming current stadiums and exposition halls into medical facilities (8). FSHs played an essential role in increasing admission capacity, treating infected patients under mild or moderate conditions, isolating confirmed and asymptomatic cases, and blocking community spread (9).

Sudden public health events were previously reported predisposing individuals to a psychological crisis, with temporary failure of conventional coping strategies accompanied by mental dysfunction (10, 11). Healthcare workers have become the leading force in this battle against COVID-19, and nursing professionals, who account for more than half of them, hold the front line of prevention and treatment of NCP. Studies at home and abroad have shown that in the face of work-related stress, most people experience adverse psychosomatic reactions, leading to depression and low work efficiency (12, 13). Due to high occupational risks of infection, features of the working environment, and requirements of occupational protection, the stress on nursing professionals is much higher than on other healthcare workers (14). Although most FSH nurses received psychological training beforehand, they were more likely than other frontline or non-frontline personnel to report psychological issues (15).

Studies have confirmed that stress has a functional relationship with nurses' work adaptation (16, 17). It is also one of the external factors affecting the quality of life. Previous studies have shown that nurses' stress is mainly caused by the death of patients, conflicts with physicians, lack of support, inadequate knowledge base, heavy workload, conflicts with other colleagues, insufficient knowledge, low social status, low financial income, and lack of job autonomy (18–21). Moderate stress has a motivational effect and promotes resilience, enabling nurses to cope with work-life challenges (22). Conversely, excessive stress could negatively impact patient safety, job satisfaction, environmental adaptation (the process by which the individual balances with the environment), performance, burnout, career development, physical and mental health, and turnover intention (23–27).

Previous studies have shown that healthcare workers are among those exposed to a wide range of risks, and psychosocial risks are prevalent in this sector even during routine work (28–30). Of course, the pandemic might amplify the underlying risk factors (31). During the pandemic, studies on negative emotions among healthcare workers were widely conducted, especially among nurses (32, 33). The high risk of infection, excessive workload, unsafe working environment, increased number of confirmed and suspected cases, negative patient emotions, lack of touch with family members, and a social context with uncertainty and conflicts might contribute to their stress (34–36). Frequent night shifts, fatigue, fear of infection, overwork, and self-blame for patients' adverse outcomes have been proven factors for frontline healthcare workers' stress load (35, 36). Additionally, researchers have emphasized the necessity to concentrate on the work-related stress experienced by frontline healthcare workers during the COVID-19 outbreak (37).

Psychosocial risk factors in workplaces and their impact on health and the economy have become one of the most challenging issues in the field of occupational safety and health (OSH) in developed industrialized countries, and research on this issue began in the early 1960s in Europe and the United States (38). These countries have now incorporated psychosocial risk factors and workplace stress prevention and control into their national OSH regulations, such as the Occupational Safety and Health Act (1970) in the United States (39). Since the introduction of the Occupational Disease Prevention and Control Act of the People's Republic of China in 2001 (40), China's regulations and standards on OSH have been continuously improved. However, Chinese research on psychosocial risks in OSH started in the early 1990s and mainly focused on occupational stress and its effects on health (41, 42).

However, there are very few studies on FSH nurses. The few studies that have been conducted suggest that nurses may have higher levels of burnout and lower sleep quality during the pandemic (15, 43). Hence, FSH nurses' stress and mental health status during outbreak control require much attention. In addition, existing studies tend to classify subjects' psychological stress levels based only on their scores on standardized instruments [e.g., the Impact of Event Scale-6 (IES-6), the Nurse Job Stressors Scale, Perceived Stress Scale (PSS)], ignoring the heterogeneity of stress among individuals (44–46). It is a limitation of such an approach when distinguishing between group characteristics of stress and within-group differences because individuals with the same stress score may respond differently to each item. Latent profile analysis (LPA) is an individual-centered approach to determine the classification of observations based on posterior probability. LPA has been widely used in psychology, pedagogy, and other academic fields (47, 48).

Therefore, the purpose of this study was to identify FSH nurses' stress load profiles, explore their characteristics and determine influencing factors of profile membership. We hope this study would serve as a basis for early intervention and enhancement of the mental health of nurses in FSHs and motivate initiatives on developing stress-coping strategies, psychological support procedures, and a magnetic work environment.

## 2. Methods

### 2.1. Participants

This cross-sectional study was conducted in FSHs in Shanghai from March 2022 to May 2022 and included 609 nurses finally. We calculated an estimated sample size of 426 (10 times the variables) to allow for a sample loss of 15%. Initially, we selected eight FSHs with different admission scales considering the participants' representativeness and the research's feasibility. Then, we used random clustered sampling and expected to recruit 700 nurses who met the inclusion criteria. The inclusion criteria were (1) registered nurses, (2) work experiences in the red zone (contaminated area) of FSHs, and (3) informed and willing to participate. The exclusion criteria were (1) a history of mental deficiency or psychiatric diseases; (2) working in logistics or administrative positions. Finally, 78 nurses refused to participate, and 622 subjects were recruited, with a response rate of 88.86%. After the exclusion of invalid questionnaires (with the same options selected for 70% of the items or with a completion time of fewer than 2 min), 609 questionnaires were included in the final analysis, with an effective response rate of 87%.

### 2.2. Procedures

In this study, questionnaires were distributed and collected during the Omicron wave in Shanghai from March 2022 to May 2022. After contacting the nursing administrators of the FSHs and obtaining cooperation, the online survey link was distributed to them through the questionnaire web platform (wjx.cn) and then distributed to the selected nurses' WeChat groups. Participants completed a structured online questionnaire anonymously to provide information on socio-demographic data, work-related stressors, and stress load. A total of 609 questionnaires were collected. This study was reviewed and approved by the Ethics Committee of NO 905 Hospital of PLA Navy (NO.2022-17), and all the participants gave consent to complete the online survey.

### 2.3. Measures

#### 2.3.1. Socio-demographic information

This section of the questionnaire included gender, age, education, professional title, marital status, maternity status, working time in FSHs, self-perceived health condition, history of psychological training, and experience in epidemic control.

#### 2.3.2. Chinese nurses stressor scale

The Chinese Nurses Stressor Scale (CNSS) was developed by Li and Liu (49) to assess perceptions of work-related stressors with reference to nurse occupational stress research approaches proposed by Wheeler (50). After cultural adaptation and validation, the CNSS consists of 35 items divided into five dimensions covering profession development (PD), workload (WL), work environment (WE), patient care (PC), and relationship with administrators and colleagues (RAC). A Likert 5-point scale was used in our study, with “1” meaning “strongly disagree” and “5” meaning “strongly agree”. This scale has been widely used to investigate the work stressors of ICU, psychiatric, and standardized training nurses in China, with the Cronbach's alpha coefficient for the original scale being 0.94. The Cronbach's alpha coefficient of the present study was 0.97, and the coefficients of the five dimensions were above 0.8.

#### 2.3.3. Stress overload scale

Amirkhan (51) created the Stress Overload Scale (SOS), which Xi and Leilei (52) translated and culturally adapted in China. Following extensive consultation with relevant experts and stringent tests on nurses in clinical settings. The Cronbach coefficient was 0.936, the retest reliability was 0.858, and the content validity was 0.86, indicating good reliability and validity. The SOS is divided into two dimensions: Personal Vulnerability (PV, 12 items), in which people react to events that cause them to feel powerless, frail, and tired, and Event Load (EL, 10 items), in which people are subjected to extreme external events, responsibilities, and pressure. A Likert 5-point scale was used, with never = 1, rarely = 2, occasionally = 3, frequently = 4, and always = 5. The total PV score was 60, and the total EL score was 5, with higher scores in each dimension indicating greater stress. The Cronbach's alpha coefficient for the present study was 0.98.

### 2.4. Statistical analysis

We performed LPA using the R software 3.4.2 based on a set of indicators (the 22 items of the SOS) to identify the latent subgroups of FSH nurses' stress load. To determine the optimal number of subgroups, we applied the following fit indices: the Bayesian information criterion (BIC), the Akaike information criterion (AIC), the entropy test for model evaluation, and the bootstrapped likelihood ratio test (BLRT) for model comparison. Lower BIC, AIC, and entropy values indicate a better fit. The BLRT compares the differences in fitting between *k*-1 and *k* class models. The theoretical foundation for class solutions was also considered when determining the optimal number of participant classes.

The statistical software SPSS 21.0 was also applied for data analysis. Socio-demographic data were displayed in frequency and percentage. Continuous variables like the scores of the CNSS and SOS were displayed using mean and standard deviation. Comparisons of categorical variables between the potential classes of stress load were carried out using the Chi-square test, while that of continuous variables using the Kruskal–Wallis test. Finally, multinomial logistic regression was conducted to examine the potential relationship between the stress load classes and socio-demographic variables. A statistically significant difference was accepted at a *p*-value < 0.05.

## 3. Results

### 3.1. Socio-demographic characteristics of the participants

Table 1 displays the characteristics of the participants. Of the 609 participants, 584 (95.89%) were female, 25 (4.11%) were male, and the median of their age was 30 years old. Most participants (441, 72.41%) reported bachelor's degrees or above education levels, and more than half of the participants were senior or supervisor nurses. About half of the participants were married and had one or more children. Most participants had sound (37.11%) or moderate (53.20%) self-perceived health conditions. The percentages of participants who received psychological training and had experiences in epidemic control were 57.64 and 24.47%, respectively.

**Table 1:** Socio-demographic characteristics of the sample (*n* = 609).

**Variables**	**Categories**	**[M (P25, P75)]/** **[*n* (%)]**
Gender	Male	25 (4.11)
	Female	584 (95.89)
Age (year)		30 (26, 34)
Working time in FSHs (day)		22 (5, 30)
Education	Junior college	168 (27.59)
	Bachelor's degree or above	441 (72.41)
Professional title	Junior nurse	171 (28.08)
	Senior nurse	308 (50.57)
	Supervisor nurse or above	130 (21.35)
Marital status	Single	303 (49.75)
	Married	306 (50.25)
Maternity status	Childless	324 (53.20)
	One child or more	285 (46.80)
Self-perceived health	Sound	226 (37.11)
condition	Moderate	324 (53.20)
	Out of condition	59 (9.69)
History of psychological	Yes	351 (57.64)
training	No	258 (42.36)
Experiences in epidemic	Yes	149 (24.47)
control	No	460 (75.53)

### 3.2. Latent profiles analysis of stress load

**Figure 1:**
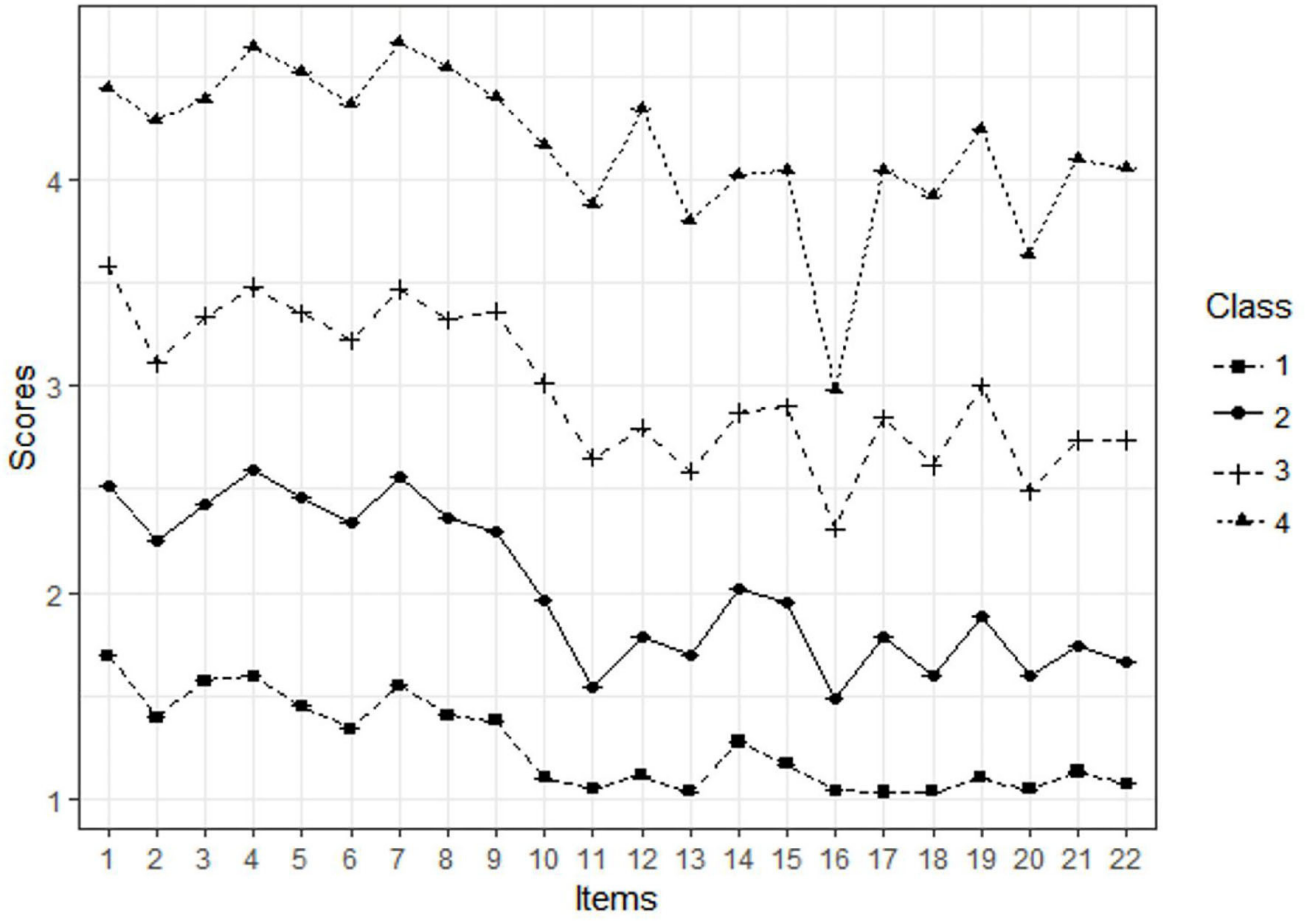
The distribution of four potential classes of stress load.

We extracted and compared the two- to five-class model solutions to classify and identify the optimal model. When comparing the models, the smaller the AIC and BIC indices, the higher the Entropy index, and the BLRT < 0.05, the better the model fit. As seen in Table 2, the 4-category model had the highest Entropy index and the second lowest BIC index, while the AIC index and the *p*-value of BLRT reached a significant level, thus making it the best model, with 232 (38.1%) in Class 1, 214 (35.1%) in Class 2, 122 (20.0%) in Class 3, and 41 (6.7%) in Class 4. Figure 1 illustrates the distribution of the potential stress load classes based on the 22 items of SOS. The *x*-axis of Figure 1 means the 22 items of the Stress Overload Scale (SOS), and the *y*-axis means the average score of each item. Thus, Figure 1 gives a snapshot of stress load levels across the four classes.

**Table 2:** Model fit indices of latent profile analysis of stress load (*n* = 609).

**Model**	**AIC**	**BIC**	**Entropy**	**BLRT**	**Proportion of the least class**
2-class	30,409.028	30,704.620	0.988	0.010	28.2%
3-class	27,632.107	28,029.171	0.965	0.010	19.9%
**4-class**	**25,759.536**	**26,258.072**	**0.974**	**0.010**	**6.7%**
5-class	25,160.237	25,760.244	0.956	0.010	5.6%

AIC, Akaike Information Criterion; BIC, Bayesian Information Criterion; BLRT, bootstrap likelihood ratio test.

Bold values means the selected optimal model of the latent profile analysis.

### 3.3. Characteristics of classes

Table 3 presents the PV and EL scores reflecting each class's stress load. Class one had the highest proportion of the sample, 38.1% (232/609), and was labeled “low stress load class.” Low SOS scores in this class indicated light stress in participants. Class two, “mild stress load class,” comprised 35.1% (214/609), showing relatively mild stress. Class three, “moderate stress load class,” made up 20% (122/609) of the sample, while Class four, “high stress load class,” had the lowest proportion, 6.7% (41/609), indicating that participants in this class had the highest level of stress load among the four classes [*M* (P25, P75) = 89 (84.5, 99)].

**Table 3:** SOS scores for different stress load classes (*n* = 609).

**Dimensions**	**Class 1** ** (*n* = 232)** ** [*M* (P25, P75)]**	**Class 2** ** (*n* = 214)** ** [*M* (P25, P75)]**	**Class 3** ** (*n* = 122)** ** [*M* (P25, P75)]**	**Class 4** ** (*n* = 41)** ** [*M* (P25, P75)]**	**Total sample** ** (*n* = 609)** ** [*M* (P25, P75)]**
Personal vulnerability	16 (13, 20)	27 (24, 30)	38 (36, 41)	52 (49, 57.5)	24 (19, 35)
Event load	10 (10, 12)	18 (15, 20)	28 (24, 30)	39 (35, 42)	16 (11, 22)
Total score	27 (23, 32)	44 (40, 48.25)	66 (61, 70)	89 (84.5, 99)	30 (42, 57)

Class one: “low stress load”; Class two: “mild stress load”; Class three: “moderate stress load”; Class four: “high stress load”.

**Figure 2:**
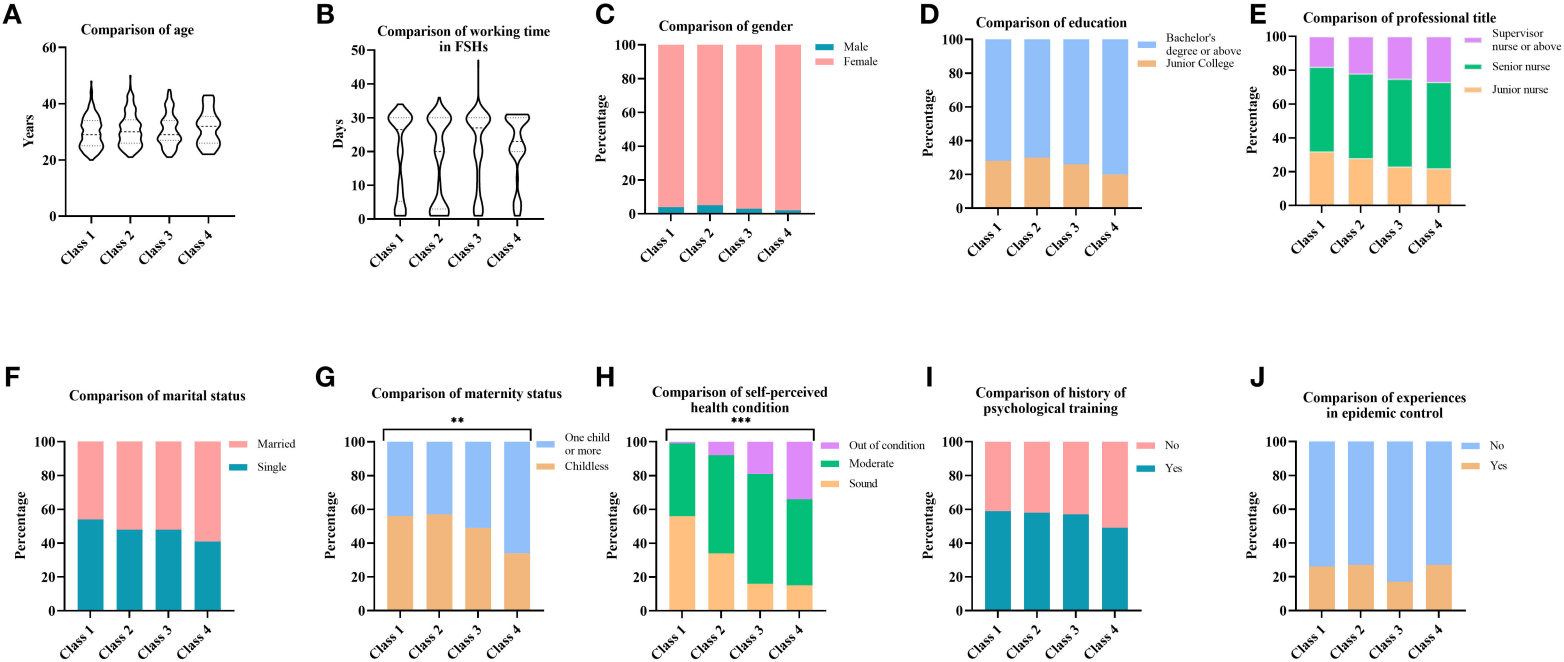
**(A–J)** Characteristics of individuals in potential stress load classes. ***p < 0.001, **p < 0.01.

Figure 2 provides the socio-demographic characteristics of the participants in each class and their perception of work-related stressors. The characteristics of each class were compared by the Chi-square test and the Kruskal–Wallis test, as shown in Figure 2. Regarding socio-demographic characteristics, except for maternity status and self-perceived health condition, we did not find significant differences in gender, age, education, professional title, marital status, working time in FSHs, history of psychological training, and experience in epidemic control among the four classes.

### 3.4. Multinomial logistics regression

The results of the multinational logistic regression results are shown in Table 4. The predictor variables were age, education, maternity status, working time in FSHs, and the five dimensions of work-related stressors (PD, WL, WE, PC, and RAC), with class 1 as the reference group during the analysis. Compared with Class 1, participants who were senior (OR =1.091, 95% CI: 1.023, 1.163), had children (OR = 4.169, 95% CI: 1.855, 9.368), perceived unhealthy (OR = 0.207, 95% CI: 0.059, 0.722), worked longer time in FSHs (OR = 3.765, 95% CI: 1.838, 7.714), faced challenges toward patient care (OR = 1.900, 95% CI: 1.013, 3.563) and had poorer working relationships (OR = 2.147, 95% CI: 1.078, 4.272) were more likely to enter Class 2. Furthermore, participants tended to be grouped into Class 3 if they had bachelor's degrees or above (OR =1.050, 95% CI: 1.019, 1.081), had children (OR = 3.447, 95% CI: 1.316, 9.030), perceived unhealthy (OR = 0.099, 95% CI: 0.029, 0.340), worked longer time in FSHs (OR = 7.071, 95% CI: 2.978, 16.790), faced with more workload (OR = 2.665, 95% CI: 1.140, 6.232), and worse working environment (OR = 8.922, 95% CI: 2.893, 27.513). Finally, those perceived unhealthy (OR = 0.045, 95% CI: 0.012, 0.171), worked longer time in FSHs (OR = 40.483, 95% CI: 12.103, 135.410), faced with more workload (OR = 3.664, 95% CI: 1.047, 12.815), and worse working environment (OR = 12.274, 95% CI: 3.029, 49.729) were more likely to be assigned to Class 4.

**Table 4:** Multinomial logistic regression on stress load classes (*n* = 609).

**Variables**	**Class 2**		**Class 3**		**Class 4**	
	***b***	**OR (95% CI)**	***b***	**OR (95% CI)**	***b***	**OR (95% CI)**
Age	0.087^**^	1.091 [1.023, 1.163]	0.038	1.039 [0.951, 1.135]	0.065	1.068 [0.934, 1.220]
Education	0.012	1.013 [0.993, 1.033]	0.048^**^	1.050 [1.019, 1.081]	0.048	1.049 [0.995, 1.106]
Maternity status	1.428^**^	4.169 [1.855, 9.368]	1.238^*^	3.447 [1.316, 9.030]	0.914	2.494 [0.667, 9.321]
Self-perceived health condition	1.792^**^	0.207 [0.059, 0.722]	2.079^***^	0.099 [0.029, 0.340]	1.540^*^	0.045 [0.012, 0.171]
Working time in FSHs	1.326^***^	3.765 [1.838, 7.714]	1.956^***^	7.071 [2.978, 16.790]	3.701^***^	40.483 [12.103, 135.410]
Profession development	0.039	1.040 [0.582, 1.858]	0.390	1.477 [0.738, 2.956]	0.733	2.082 [0.861, 5.031]
Workload	0.665	1.945 [0.962, 3.930]	0.980^*^	2.665 [1.140, 6.232]	1.298^*^	3.664 [1.047, 12.815]
Environment	0.600	1.821 [0.678, 4.895]	2.189^***^	8.922 [2.893, 27.513]	2.507^***^	12.274 [3.029, 49.729]
Care for patients	0.642^*^	1.900 [1.013, 3.563]	0.491	1.634 [0.682, 3.913]	0.802	2.230 [0.521, 9.551]
Relation with colleagues	0.764^*^	2.147 [1.078, 4.272]	0.207	1.230 [0.479, 3.163]	−0.347	0.707 [0.165, 3.032]

^***^p < 0.001,

^**^p < 0.01,

^*^p < 0.05.

Reference group: class one; OR: odds ratio; 95%CI: 95% confidence interval.

## 4. Discussion

This cross-sectional study investigated the stress load profiles of 609 nurses in FSHs and determined their characteristics and influencing factors. A total of four classes of stress load were identified through LPA, labeling as low (Class 1, 38.1%), mild (Class 2, 35.1%), moderate (Class 3, 20.0%), and high (Class 4, 6.7%) stress load. The FSH nurses' relatively low median score of SOS [*M* (P25, P75) = 30 (42, 57)] implied that most participants in our study underwent modest stress load during their working in FSHs. Through comparison analysis, maternity status and self-perceived health condition were significantly different among participants in the four classes. Furthermore, the influencing factors of the stress load profiles were determined as age, education, maternity status, working time in FSHs, and the four dimensions of work-related stressors (WL, WE, PC, and RAC). Participants who perceived less healthy, worked longer in FSHs, faced more workload, and had a worse working environment were more likely to be grouped into the high stress load class.

In contrast to previous reports, nurses in this study had less stress load, while health care workers were formerly assessed at a moderate to high stress level during the pandemic's initial stage. For instance, Murat et al. (46) found that nurses in Turkey experienced high levels of stress and moderate levels of depression during the pandemic outbreak. Shahrour and Dardas (53) found that 64% of Jordanian nurses experienced acute stress disorder during the initial phase of the pandemic, and 41% experienced psychological distress. Furthermore, Ahn et al.'s (35) team reported high work-related stress and anxiety to COVID-19 among healthcare workers in South Korea in April 2020, especially nursing professionals who are single. Similar circumstances occurred during the pandemic in Latin American nations, where one-third of healthcare workers were estimated to experience acute stress (36). This finding might be due to the rapid transmission of the epidemic, inadequate staffing, lack of awareness of the NCP, and psychological resilience in the early stages.

The LPA results showed that the stress load of FSH nurses could be divided into four classes, with Class 1 and Class 2 accounting for a total of 73.2%, indicating that the overall stress load of FSH nurses was at a modest level. As is known, occupational role, training/preparedness, high-risk work conditions, quarantine, role-related stressors, perceived risk, social support, social rejection/isolation, and the effect of diseases on personal lives were linked to the psychological health of healthcare workers (54). Administrators in FSHs recognized that management of occupational safety and health is essential and took action. Therefore, this finding might be attributed to the fact that FSHs had comprehensive preparation regarding the overall layout, work environment, work procedure, knowledge training, and supply of protective equipment.

Regarding the characteristics of FSH nurses' stress load profiles, participants in the four classes significantly differed in maternity status and self-perceived health condition. Participants who had children and perceived less healthy were more likely to be grouped into classes of higher stress load. This finding is in accord with Tahara's (55) research suggesting that good health status is associated with a reduced risk of mental health problems. However, in contrast to Vahedian-Azimi's (56) results on stress among critical care nurses, the number of children was not significantly associated with stress levels, which could be attributed to the differences in context and setting between the studies. This serves as a reminder to administrators to consider the health and maternity status of FSH nurses when recruiting frontline caregivers to participate in the fight against NCP. It is recommended that health check-ups be conducted before going to the frontline, that those in good health be selected, and that immunization-enhancing interventions be given as appropriate. For frontline personnel with heavy family burdens, individuals are suggested to seek social support and undergo a regular psychological assessment. Additionally, organizations should develop appropriate support mechanisms to help resolve challenges faced by healthcare workers and provide a safe working environment to safeguard their physical and emotional well-being.

Alarmingly, 75.53% of the FSH nurses had no experience in supporting the front line of prevention and control of COVID-19. Unlike Osman's (57) study on stigma and worry perceptions among Egyptian healthcare providers from contracting COVID-19 infection, there was no difference in epidemic prevention and control experience in stress load classes. Even so, we suggest that medical institutions gradually establish a comprehensive training system for nursing emergency human resources, organize and implement drills based on the COVID-19 outbreak, and reserve many professional nursing emergency rescue teams for epidemic prevention and control.

Concerning the influencing factors for stress load profile membership, the present study found that FSHs nurses who were senior, had children, worked longer time in FSHs, faced challenges toward patient care, and interpersonal relationships were more likely to be classified into mild stress load (Class 2). Meanwhile, participants tended to be grouped into moderate stress load (Class 3) if they were undergraduates, had children, worked longer time in FSHs, or faced more workload and worse working environment. Alarmingly, those who worked longer in FSHs, faced with more workload and a worse working environment, were prone to high stress load (Class 4). Likewise, Zhan et al.'s (45) survey on job stress among frontline nurses fighting COVID-19 showed that nurses with higher seniority and educational level had higher job stress. One explanation might be the higher expectations of work and sense of responsibility among nurses with higher seniority and educational level. Additionally, this study found that maternity status impacted the FSH nurses' stress load profile membership, similar to earlier studies reporting that nurses concerned for families were susceptible to psychological distress (58, 59). Therefore, during the prevention and control of COVID-19, administrators need to dedicate themselves to caring for the families of FSH nurses and providing psychological support. At the same time, nursing professionals are encouraged to communicate more with their families to reduce and eliminate unnecessary barriers.

Moreover, work procedures in FSHs are complex, and conflicts with patients during care occasionally occur, which might lead to increased psychological pressure on nurses. The high workload of FSH nurses strains the workforce and leads to stress. Studies have shown that working time and workload positively correlate with mental distress (60). Other work-related stressors like WE and RAC are also worth discussing as FSH nurses are constantly faced with various tasks, isolation requirements, personal protective stress, and unknown risks. Consistent with Firew's findings (61), the more unknown and complex risks in the work environment than expected, the more negative psychological and physical outcomes for healthcare workers. Therefore, it is essential to clarify the scope of each position, scientifically allocate human resources, and adjust the nursing staff structure dynamically in the FSH nursing management. Noteworthy, feeling valued by organizations helps to eliminate stress, as mentioned previously by (62). Therefore, an emergency nursing management system for major infectious disease epidemics should create a professional emergency nursing team to ensure human resource deployment. Affirmation, encouragement, and respect from administrators could positively impact FSH nurses and enhance their sense of pride and belonging. At the same time, support and understanding among colleagues can help nurses gain social support and help them to be more committed to their careers (63).

Accordingly, policymakers and nursing administrators should pay close attention to the work stress of frontline nursing professionals while carrying out the fight against the pandemic. Taking active and effective interventions and psychological support for FSH nurses might help to have a positive mindset and ensure a regular clinical routine. At the governmental level, occupational psychosocial risks should be included in the scope of OSH, including regulations, policies, and standards. At the organizational level, administrators are encouraged to work on preventing and controlling psychosocial risks and promoting mental health in workplaces. At the individual level, healthcare workers might increase awareness through universal training in psychosocial risk coping strategies.

## 5. Limitations

Despite our efforts to make this study scientifically rigorous, we should be mindful of the several limitations of this study. Owing to a cross-sectional design, this study lacks a controlled sample distribution and has a degree of non-response bias, which might affect the sample's representativeness and the findings' generalisability. Furthermore, due to the need for epidemic prevention and control, the surveyors could not contact the respondents in person, so the survey was conducted on voluntary participation, and self-assessment questionnaires were used. Some FSH nurses with mental health problems may have been omitted from the survey or concealed their mental problems in the questionnaire. Meanwhile, the responses relating to self-rated work stressors and stress load were subjective and were, therefore, susceptible to recall bias. Finally, given the probability of second psychological trauma during the process of the stress survey, there may be a need for further investigation and validation using post-traumatic growth scales.

## 6. Conclusions

Nursing professionals might have cognitive, emotional, and behavioral changes as a result of working in FSHs. In this study, the stress load of FSH nurses was classified into four classes by LPA, and only 6.7% of participants were assigned to high stress load (Class 4), indicating that most participants were at a low stress level. The stress load profiles of nurses in different classes were well differentiated. Factors influencing stress load profiles include age, education, maternity status, working time in FSHs, and the four dimensions of work-related stressors (WL, WE, PC, and RAC). This finding suggests that we should develop different psychological training programs according to the potential class of FSH nurses to improve their stress resilience and adaptability in emergencies and to help nurses channel their stress rationally. Particular attention should also be paid to participants who worked longer time in FSHs, were faced with more workload, and had a worse working environment.

## Data availability statement

The raw data supporting the conclusions of this article will be made available by the authors, without undue reservation.

## Ethics statement

The studies involving human participants were reviewed and approved by the Ethics Committee of 905th Hospital of the PLA Navy. The patients/participants provided their informed consent through online survey links to participate in this study.

## Author contributions

JC and JW: conceptualization, supervision, and funding acquisition. LG, PF, and HX: investigation and data collection. LG and JC: statistical analysis and writing of the paper. LG, JC, JW, and HX: revision and editing of the paper. All authors contributed to the article and approved the submitted manuscript.
